# Supplementary material for: Unraveling the Intertwined Effect of pH on Helicobacter pylori Motility and the Microrheology of the Mucin-Based Medium It Swims in
Source: Microorganisms. 2023 Nov 10;11(11):2745. doi: 10.3390/microorganisms11112745 (PMC10673263; doi:10.3390/microorganisms11112745)
Supplement: Supplementary file 1 [file microorganisms-11-02745-s001.zip › Supplementary Materials-revised 10-15-2023.pdf]

Supplementary Materials

Text and Figure S1: pH-dependent microrheology of BB10 and PGM

Methods: To measure the microrheology of BB10 and PGM at various pH, we tracked several hundred to thousand trajectories from the Brownian motion of micron-size polystyrene latex particles suspended in the solutions using time-resolved fluorescence microscopy. The mean square displacement (MSD) versus time was calculated for each particle trajectory. The viscosity, η, was computed as detailed in Su et al. [25] using the equation for the time dependence of MSD for the 2-dimensional diffusive motion of particles,

$$MSD(t) = 4D_o t \quad \text{(S1)}$$

where D_o is the diffusion constant. The viscosity is inversely proportional to D_o via the Stokes-Einstein relation,

$$\eta = \frac{kT}{6\pi r D_o} \quad \text{(S2)}$$

k is the Boltzmann constant, T is the temperature in Kelvin, and r is the radius of the particle.

Results: Fig. S1**A** shows the $<MSD>$ averaged over all particles in BB10 and PGM at various pH on a log-log plot, with water as a reference. We found that the $<MSD>$ and the viscosity of BB10 are similar to those of water, and not pH-dependent (Fig. S1**A**, **C**). In contrast in PGM, it is evident from the log-log plot of $<MSD>$ that equation 1 is no longer valid and instead MSD is proportional to t^{α}. By fitting the $<MSD>$ *vs* time we obtained the exponent $\alpha < 1$, indicating that the mobility of the microparticles embedded in PGM is sub-diffusive, i.e. hindered at low pH as a result of the sol-gel transition that PGM undergoes as pH decreases below 4 [14,27]. As pH decreased from 6.7 to 3.7 in PGM, α decreased from 0.8 to 0.6, implying increasing sub-diffusivity of particles in PGM as it gels (Fig. S1**B**). In comparison to PGM, the microparticles in BB10 showed normal diffusion with α = 1 (Fig. S1**B**). Fig. S1**C** shows the viscosities of BB10 and 15mg/ml PGM calculated using Eqn. (S2). In the case of PGM the effective viscosity was estimated by using only the data in the long-time regime where $\alpha \sim 1$. PGM is about 50 times more viscous than BB10 at pH 6, and the viscosity of PGM increased rapidly, by a factor of 2, as pH decreases from pH 6.1 to 2, whereas the viscosity of BB10 remains constant as pH decreases (Fig. S1**C**). Furthermore, in PGM the exponent α is time-dependent, varying between 0.5 to 1, reflecting the frequency dependence of the moduli of the viscoelastic PGM [14, 16, 27]. The complex viscoelastic moduli of PGM as a function of pH are reported in Constantino [27]. The viscosity estimated from the viscoelastic moduli agreed with that obtained from the MSD within 10%.

We evaluate the effect of gelation on mucin by computing *HR* defined by the ensemble-averaged variance and the mean of *MSD*,

$$HR(\Delta\tau) = \frac{var(MSD(\Delta\tau))}{<MSD(\Delta\tau)>^2} \quad \text{(S3)}$$

Here $\Delta\tau$ is the characteristic lag time. This parameter can be taken as a measure of spatial heterogeneity in a non-homogeneous system. In particle tracking measurements due to particles constantly diffusing in and out of the focal plane, the ensemble averaging of *MSD* across all particles in a medium with spatially heterogeneous rheology tends to result in a statistical bias toward more motile particles, as they have a higher probability of leaving and re-entering the field of view, producing segmented, shorter tracks. By calculating *HR*, each trajectory is weighted by a factor proportional to its duration in time. Fig. S1 C shows the time-averaged <HR> along with its standard deviation as a function of pH in BB10 and PGM. We found that HR_{BB10} ~ 0.1 at all pHs measured, consistent with BB10 behaving like a watery Newtonian fluid, whereas at $\Delta\tau$ = 0.1s HR_{PGM} increases from ~0.5 to nearly 1 as pH decreased from 6.7 to 3.7 indicative of increasing heterogeneity as pH decreases. The *HR* data in PGM shows a peak at pH 5, we suspect that this reflects a biphasic behavior of *HR*, with two characteristic trends corresponding to the gel phase at pH 4 and lower and the solution phase at pH 5 and above. From AFM measurements [17] we have shown that PGM fibers which are uniformly distributed at pH 6 begin to aggregate at pH 5, perhaps this leads to an increase in <HR> as particles encounter varying polymer concentrations in different regions. In the gel phase, the particles are excluded from the regions with a higher concentration of fiber bundles and exhibit hindered motion confined in the pores of the gel. In this case, the inhomogeneity will depend on the extent of crosslinking and the degree of gel swelling/shrinking.

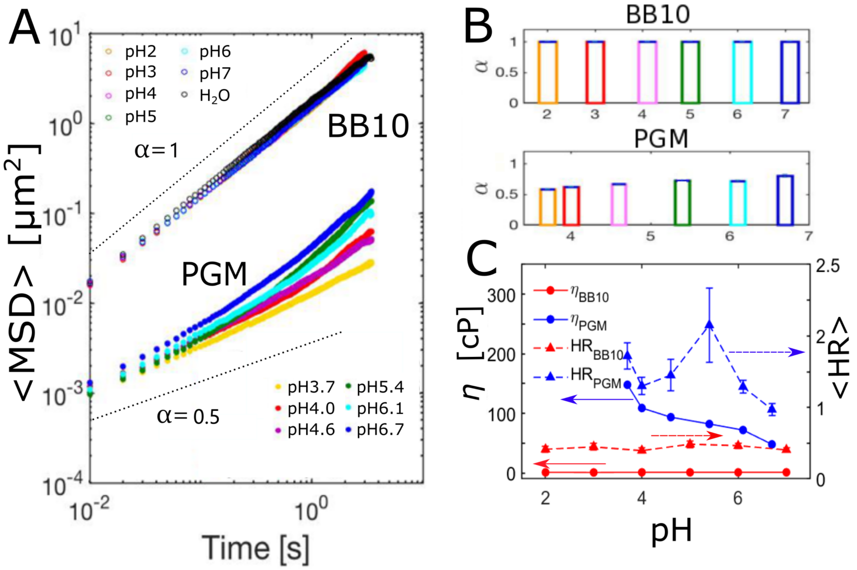

Figure S1. Microrheology of BB10 and PGM at various pHs. (**A**) Average mean-square displacement (<*MSD*>) of BB10 (open symbols), and PGM (filled symbols) at pH 2 - 7 averaged over all particles. (**B**) Bar graphs of the exponent α *versus* pH calculated from <*MSD*> of all particles. Lines with slope α = 1 and 0.5 are drawn to guide the reader. (**C**) <*HR* > and viscosity η of BB10 and 15 mg/ml PGM calculated as discussed in the text. The arrows in (**C**) point toward the respective axes.

Text and Figure S2: Probability distribution $p(d, \tau)$ of bacteria swimming

Fig. S2 shows a contour plot of the probability p (d, τ), where the contours represent curves of equal probability. Some characteristics of the differences in swimming between BB10 and PGM are obvious from this plot. The figure illustrates that the bacteria swim for a much longer time in the image plane in PGM as compared to BB10, implying that the bacteria swim faster in BB10 as compared to PGM. In both media, the probability to swim to larger distances is reduced with decreasing pH. In BB10 this decrease occurs around a pH of 5, whereas in PGM no trajectories extend beyond 20 μm at a pH of 4, reflecting the gelation of PGM close to pH 4.

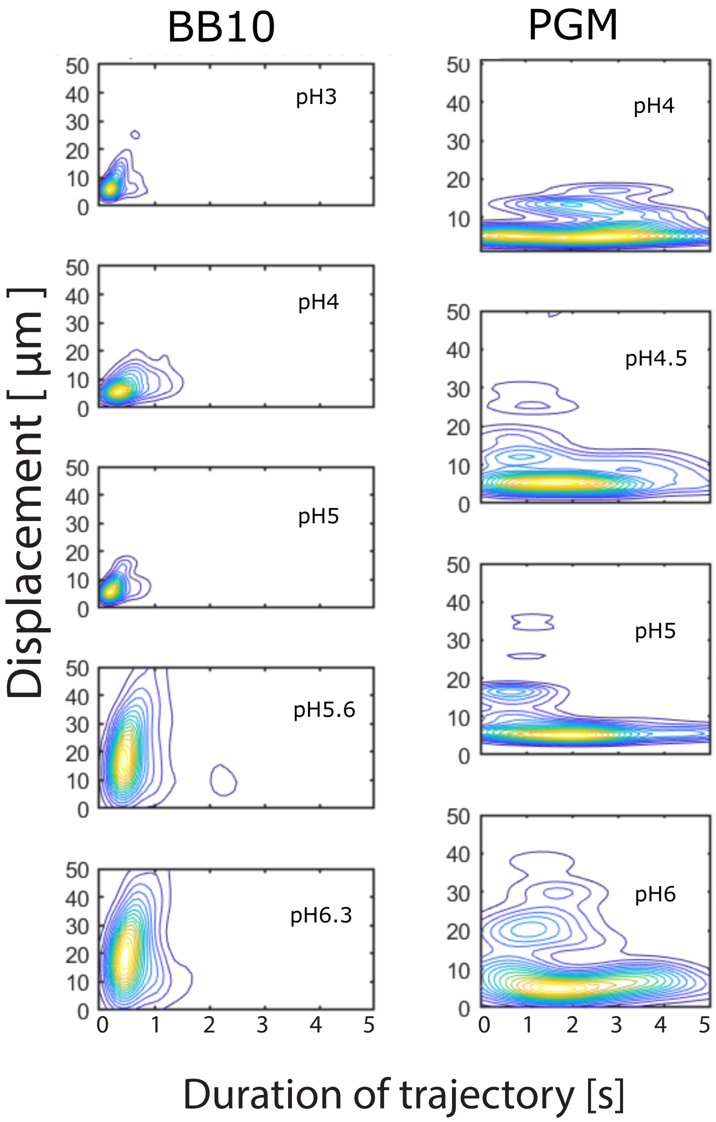

Figure S2. Contour plots of probability $p(d, \tau)$ for *H. pylori* swimming in BB10 or PGM at different pHs as indicated. Each curve corresponds to a fixed value of the probability, with the highest probability on the plot displayed in brighter colors.

Videos

Video S1. Phase contrast microscopic video of a bacterium at 100X swimming in BB10 at pH 4, showing forward and reverse motions, as described in the text.

Video S2. Phase contrast microscopic video of a bacterium rotating about a fixed point in PGM gel at pH 4 with slow and fast body rotation rates over time.

Video S3. Phase contrast microscopic video of a bacterium translating in a fixed circular trajectory in PGM at pH 4.

Video S4. Phase contrast microscopic video of a bacterium in PGM at pH 4.5 showing rotations with random translational motions.

Video S5. Phase contrast microscopic video of a bacterium in BB10 at pH 4 showing translational displacement while rotating.
